# Supplementary material for: Early Life Antimicrobial Exposure: Impact on Clostridioides difficile Colonization in Infants
Source: Antibiotics (Basel). 2022 Jul 21;11(7):981. doi: 10.3390/antibiotics11070981 (PMC9311587; doi:10.3390/antibiotics11070981)
Supplement: Supplementary file 1 [file antibiotics-11-00981-s001.zip › antibiotics-1788484-supplementary.pdf]

antibiotics

Supplementary File

Table S1. Distribution (%) of antibiotic exposure, household cleaning products use and selected covariates at 3–4 months, 12 months and both time points; compared to the entire CHILD cohort at 3 sites (Edmonton, Winnipeg, Vancouver).

Column percentages	3–4 Month Sample % *n*=1,429	12 Month Sample % *n*=1,728	Persistent Sample** % *n*=653	CHILD Cohort at 3 Sites % *n*=2,150
**Any antibiotics***				
No	51	50	52	50
Yes	49	50	48	50
**Household cleaning products use**				
Lower (below average)	53	54	54	53
Higher (above average)	47	46	46	47
**Birth method**				
Vaginal	78	75	77	76
Caesarean	22	25	23	24
**Breastfeeding**				
No	18	14	16	15
Yes	82	86	84	85
**Infant sex**				
Male	54	52	56	52
Female	46	48	44	48
**Smoke exposure**				
No	82	83	83	82
Yes	18	17	16	18

Notes: *Any antibiotics: Exposure to maternal intrapartum antibiotic or direct infant antibiotic by 3 Months. **Persistent sample: Subset of infants used to identify *C. difficile* colonization at both 3–4 months and 12 months (persistent colonization).

Table S2. Sensitivity analysis for antimicrobial exposure and *C. difficile* colonization at 12 months of age.

	Adjusted OR† (95% CI)	*p* value	Adjusted OR‡ (95% CI)	*p* value
**Antimicrobial Exposure* (ref=NALC)**				
AALC	1.35 (1.00-1.83)	**0.043**	1.41 (1.02-1.94)	**0.034**
NAHC	1.12 (0.84-1.49)	0.438	1.06 (0.78-1.44)	0.694
AAHC	1.35 (0.99-1.85)	0.057	1.33 (0.95-1.88)	0.095

Notes: *Antimicrobial exposure by 3 months (NALC: no antibiotics and lower usage of cleaning products, AALC: any antibiotics and lower usage of cleaning products, NAHC: no antibiotics and higher usage of cleaning products, AAHC: any antibiotics and higher usage of cleaning products); †Adjusted for antibiotics after 3 months and variables in final model, ‡ Excluding any infant that

received antibiotics after 3 months and adjusted for variables in final model; OR: odds ratio; CI: confidence interval; statistically significant *p*-values displayed in **bold.**

Table S3. Population characteristics and *C. difficile* colonization at 3–4 months and 12 months.

	C. difficile Colonization at 3–4 Months Total *n*=1429			*C. difficile* Colonization at 12 Months Total *n*=1728		
**Row percentages**	**Total[a]**	***C. difficile* Yes, *n* (%)**	***p* value (X^2 exact)**	**Total[a]**	***C. difficile* Yes, *n* (%)**	***p* value (X^2 exact)**
**Antimicrobial exposure***						
NALC	406	97 (24%)	**<0.001**	488	198 (41%)	**0.009**
AALC	346	105 (30%)		447	223 (50%)	
NAHC	318	103 (32%)		369	164 (44%)	
AAHC	359	140 (39%)		424	212 (50%)	
**Maternal age**						
18 – 29	503	195 (39%)	**<0.001**	576	268 (47%)	0.916
30 – 39	874	238 (27%)		1,094	501 (46%)	
≥40	52	12 (23%)		58	28 (48%)	
**Maternal race**						
Caucasian	1,072	346 (32%)	0.162	1315	583 (44%)	**<0.001**
Asian	203	52 (26%)		248	147 (59%)	
Other	141	42 (30%)		156	63 (40%)	
**Family income**						
<50,000	199	71 (36%)	0.201	212	85 (40%)	0.208
50,000 – 99,999	487	158 (32%)		597	271 (45%)	
≥100,000	516	160 (28%)		730	347 (48%)	
Prefer not to answer	130	39 (30%)		147	73 (50%)	
**Birth method**						
Vaginal	1,096	314 (29%)	**0.002**	1297	584 (45%)	0.248
CS-elective	134	50 (37%)		175	86 (49%)	
CS-emergency	194	77 (40%)		252	126 (50%)	
**Gestational age**						
<39 weeks	372	115 (31%)	1.000	455	226 (50%)	0.070
≥39 weeks	1,057	326 (31%)		1,267	566 (45%)	
**Infant Sex**						
Male	766	255 (33%)	0.067	896	378 (42%)	**0.001**
Female	663	190 (29%)		832	419 (50%)	
**Breastfeeding at 3 Months**						
Exclusive	791	182 (23%)	**<0.001**	1072	488 (46%)	0.717
Mixed	384	142 (37%)		420	201 (48%)	
Formula	251	121 (48%)		435	108 (46%)	
**Furry pet**						
No	777	217 (28%)	**0.005**	936	443 (47%)	0.244
Yes	648	226 (35%)		787	350 (44%)	
**Older sibling**						
No	712	240 (34%)	**0.034**	884	462 (52%)	**<0.001**
Yes	712	202 (28%)		839	333 (40%)	
**Smoke exposure**						
No	1,160	332 (29%)	**<0.001**	1,422	657 (46%)	0.697
Yes	248	103 (42%)		285	128 (45%)	

Notes: *Antimicrobial exposure by 3 months (NALC: no antibiotics and lower usage of cleaning products, AALC: any antibiotics and lower usage of cleaning products, NAHC: no antibiotics and higher usage of cleaning products, AAHC: any antibiotics and higher usage of cleaning products); [a]Total may not add up due to missing data; *p*-value calculated using Fisher's (X^2) exact test and displayed in **bold** when statistically significant.

Table S4. Individual adjustment for covariates on antimicrobial exposure and *C. difficile* colonization.

		***C. difficile* Colonization (3–4 months)**		***C. difficile* Colonization (12 months)**		**Persistent *C. difficile* Colonization**	
		**Odds Ratio (95% CI)**	***p* value**	**Odds Ratio (95% CI)**	***p* value**	**Odds Ratio (95% CI)**	***p* value**
**CRUDE OR**							
	**Antimicrobial Exposure* (Ref=NALC)**						
	AALC	1.38 (1.00-1.91)	**0.047**	1.45 (1.12-1.88)	**0.004**	1.75 (1.06-2.89)	**0.028**
	NAHC	1.52 (1.10-2.11)	**0.011**	1.17 (0.89-1.54)	0.256	1.59 (0.95-2.67)	0.075
	AAHC	2.03 (1.49-2.78)	**<0.001**	1.46 (1.12-1.90)	**0.004**	3.20 (1.96-5.23)	**<0.001**
**ADJUSTED FOR**							
**Maternal age**							
	AALC	1.45 (1.04-2.01)	**0.026**	1.45 (1.12-1.88)	**0.004**	1.83 (1.10-3.03)	**0.019**
	NAHC	1.47 (1.05-2.04)	**0.022**	1.17 (0.89-1.54)	0.256	1.60 (0.95-2.69)	0.073
	AAHC	2.11 (1.54-2.89)	**<0.001**	1.46 (1.12-1.90)	**0.004**	3.36 (2.05-5.52)	**<0.001**
**Maternal race**							
	AALC	1.36 (0.98-1.90)	0.060	1.42 (1.09-1.85)	**0.008**	1.78 (1.07-2.95)	**0.025**
	NAHC	1.47 (1.05-2.05)	**0.023**	1.18 (0.89-1.56)	0.227	1.55 (0.92-2.63)	0.099
	AAHC	1.95 (1.41-2.69)	**<0.001**	1.48 (1.13-1.93)	**0.003**	3.26 (1.99-5.36)	**<0.001**
**Birth method**							
	AALC	1.21 (0.85-1.71)	0.284	1.47 (1.10-1.96)	**0.008**	1.57 (0.91-2.69)	0.101
	NAHC	1.53 (1.10-2.12)	**0.011**	1.17 (0.89-1.54)	0.242	1.61 (0.96-2.69)	0.070
	AAHC	1.67 (1.17-2.40)	**0.005**	1.49 (1.10-2.01)	**0.008**	2.76 (1.56-4.87)	**<0.001**
**Gestational age**							
	AALC	1.38 (1.00-1.92)	**0.048**	1.40 (1.08-1.83)	**0.009**	1.70 (1.03-2.82)	**0.038**
	NAHC	1.53 (1.10-2.12)	**0.011**	1.16 (0.88-1.52)	0.286	1.59 (0.95-2.66)	0.077
	AAHC	2.02 (1.47-2.77)	**<0.001**	1.41 (1.08-1.84)	**0.010**	3.04 (1.86-4.98)	**<0.001**
**Infant sex**							
	AALC	1.37 (0.99-1.89)	0.055	1.47 (1.14-1.91)	**0.003**	1.77 (1.07-2.93)	**0.025**
	NAHC	1.52 (1.10-2.12)	**0.011**	1.17 (0.89-1.54)	0.249	1.59 (0.95-2.67)	0.074
	AAHC	2.01 (1.47-2.75)	**<0.001**	1.47 (1.13-1.92)	**0.004**	3.24 (1.98-5.24)	**<0.001**
**Breastfeeding at 3 Months**							
	AALC	1.34 (0.96-1.87)	0.076	1.46 (1.12-1.89)	**0.004**	1.59 (0.95-2.65)	**0.076**
	NAHC	1.29 (0.92-1.80)	0.138	1.17 (0.88-1.54)	0.265	1.36 (0.80-2.31)	0.252
	AAHC	1.76 (1.28-2.43)	**<0.001**	1.45 (1.11-1.89)	**0.005**	2.79 (1.69-4.61)	**<0.001**
**Furry pet**							
	AALC	1.36 (0.98-1.88)	0.061	1.47 (1.13-1.90)	**0.004**	1.73 (1.04-2.86)	**0.032**

	NAHC	1.45 (1.04-2.02)	**0.026**	1.19 (0.90-1.58)	0.197	1.53 (0.91-2.58)	0.104
	AAHC	1.90 (1.39-2.61)	**<0.001**	1.49 (1.14-1.95)	**0.003**	3.05 (1.86-5.01)	**<0.001**
**Older sibling**							
	AALC	1.34 (0.97-1.86)	0.073	1.38 (1.06-1.79)	**0.015**	1.52 (0.91-2.55)	0.104
	NAHC	1.54 (1.11-2.15)	**0.009**	1.18 (0.90-1.56)	0.224	1.72 (1.01-2.91)	0.043
	AAHC	1.95 (1.43-2.67)	**<0.001**	1.38 (1.06-1.80)	**0.016**	3.01 (1.82-4.96)	**<0.001**
**Smoke exposure**							
	AALC	1.37 (0.99-1.90)	0.055	1.44 (1.11-1.87)	**0.006**	1.73 (1.04-2.88)	**0.033**
	NAHC	1.44 (1.03-2.01)	**0.029**	1.15 (0.87-1.52)	0.295	1.45 (0.85-2.46)	0.165
	AAHC	1.90 (1.38-2.61)	**<0.001**	1.46 (1.12-1.90)	**0.005**	3.15 (1.92-2.45)	**<0.001**

Notes: *Antimicrobial exposure by 3 months (NALC: no antibiotics and lower usage of cleaning products, AALC: any antibiotics and lower usage of cleaning products, NAHC: no antibiotics and higher usage of cleaning products, AAHC: any antibiotics and higher usage of cleaning products); OR: odds ratio; CI: confidence interval; *p*-value calculated using logistic regression and displayed in **bold** when statistically significant.

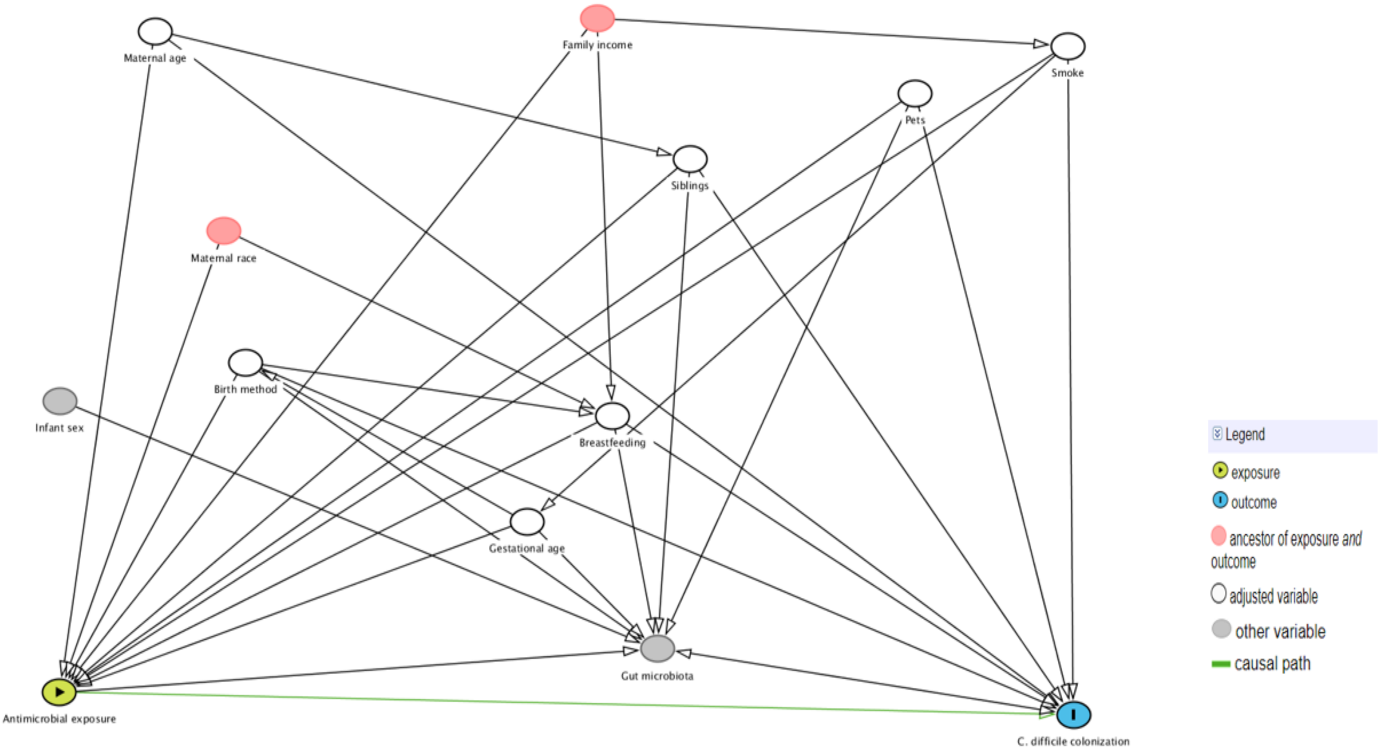

Figure S1. Directed Acyclic Graph (DAG) for antimicrobial exposure and *C. difficile* colonization at 3–4 Months (minimal sufficient adjustment sets for estimating the total effect of exposure on outcome: Maternal age, Birth method, Gestational age, Breastfeeding, Older sibling, Furry pet, Smoke exposure).

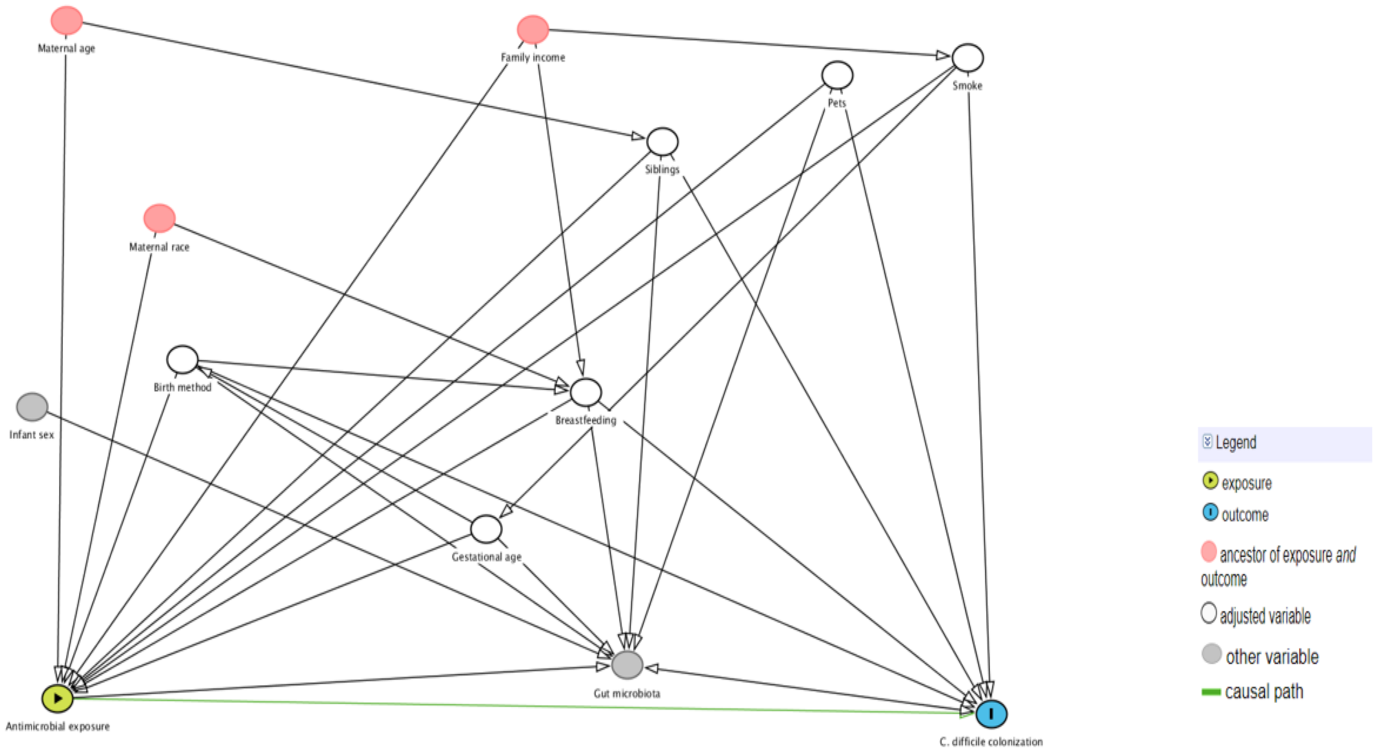

Figure S2. Directed Acyclic Graph (DAG) for antimicrobial exposure and *C. difficile* colonization at 12 Months (minimal sufficient adjustment sets for estimating the total effect of exposure on outcome: Birth method, Gestational age, Breastfeeding, Older sibling, Furry pet, Smoke exposure).

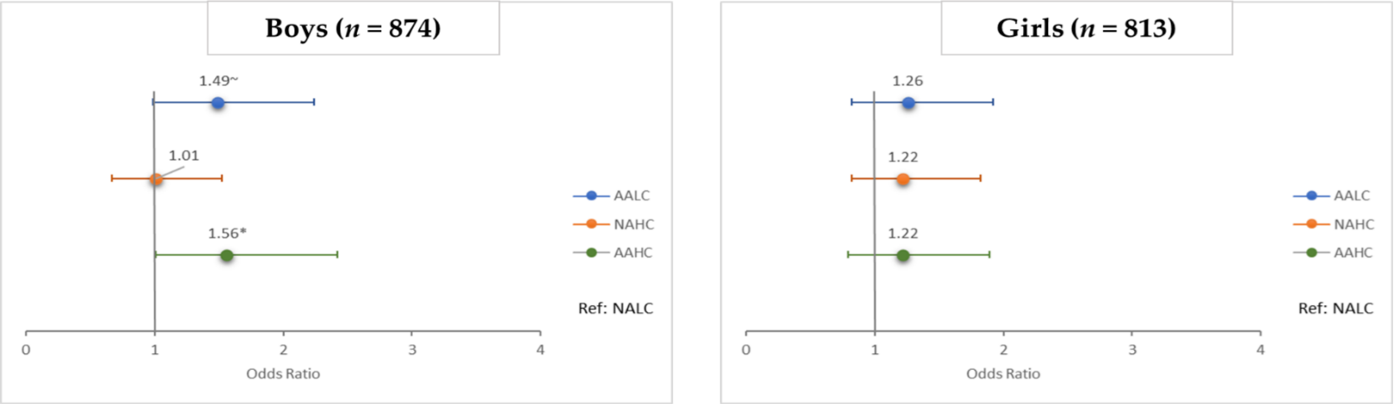

Adjusted for Breastfeeding, birth mode, siblings; ~p = 0.10; *$p < 0.05$

Adjusted for Breastfeeding, birth mode, siblings; ~p = 0.10; *$p < 0.05$

Figure S3. Stratified analysis by infant sex for antimicrobial exposure and *C. difficile* colonization at 12 months.

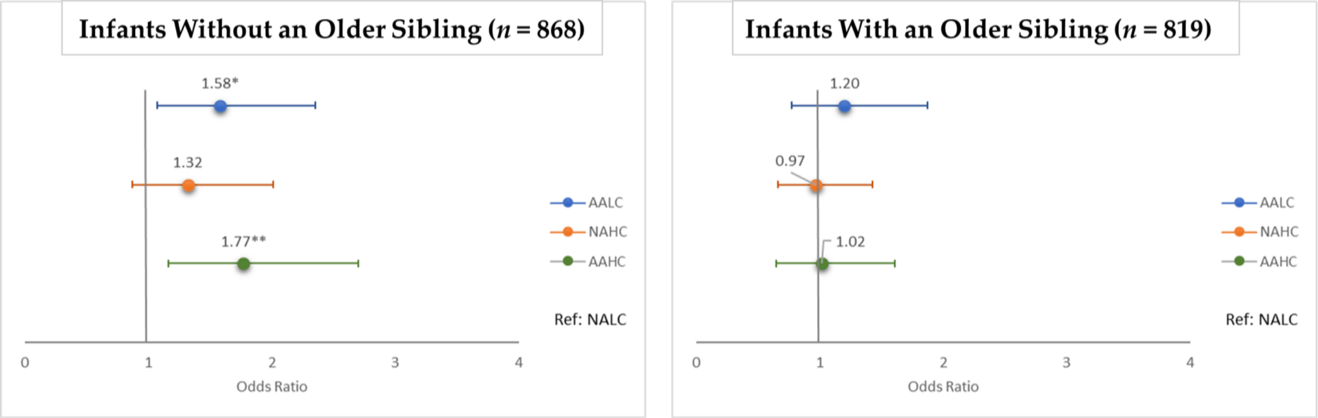

Adjusted for Breastfeeding, birth mode; ~p = 0.10; *p < 0.05; **p < 0.01

Adjusted for Breastfeeding, birth mode; ~p = 0.10; *p < 0.05; ** p < 0.01

Figure S4. Stratified analysis by older siblingship for antimicrobial exposure and *C. difficile* colonization at 12 months.

50. Which of the following products have been USED in your home since your baby was born?
(Day=daily, Wk=weekly, Mth=monthly, <Mth=less than monthly, No=not used)

50.1	Liquid or solid air freshener:	◯ Day	◯ Wk	◯ Mth	◯ <Mth	◯ No
50.2	Spray air freshener:	◯ Day	◯ Wk	◯ Mth	◯ <Mth	◯ No
50.3	Plug-in deodorizer/air freshener:	◯ Day	◯ Wk	◯ Mth	◯ <Mth	◯ No
50.4	Floor Cleaner:	◯ Day	◯ Wk	◯ Mth	◯ <Mth	◯ No
50.5	Furniture polish:	◯ Day	◯ Wk	◯ Mth	◯ <Mth	◯ No
50.6	Floor polish:	◯ Day	◯ Wk	◯ Mth	◯ <Mth	◯ No
50.7	Dusting polish or spray:	◯ Day	◯ Wk	◯ Mth	◯ <Mth	◯ No
50.8	Drain cleaner:	◯ Day	◯ Wk	◯ Mth	◯ <Mth	◯ No
50.9	Hand dishwashing detergent:	◯ Day	◯ Wk	◯ Mth	◯ <Mth	◯ No
50.10	Dishwasher detergent:	◯ Day	◯ Wk	◯ Mth	◯ <Mth	◯ No
50.11	Bleach:	◯ Day	◯ Wk	◯ Mth	◯ <Mth	◯ No
50.12	Multi-surface cleaner:	◯ Day	◯ Wk	◯ Mth	◯ <Mth	◯ No
50.13	Silver or brass polish:	◯ Day	◯ Wk	◯ Mth	◯ <Mth	◯ No
50.14	Disinfectant in bedrooms:	◯ Day	◯ Wk	◯ Mth	◯ <Mth	◯ No
50.15	Disinfectant in home in general:	◯ Day	◯ Wk	◯ Mth	◯ <Mth	◯ No
50.19	Eco & organic cleaning product:	◯ Day	◯ Wk	◯ Mth	◯ <Mth	◯ No
50.20	Glass cleaner:	◯ Day	◯ Wk	◯ Mth	◯ <Mth	◯ No
50.21	Chemical hand cleaner (e.g., for grease):	◯ Day	◯ Wk	◯ Mth	◯ <Mth	◯ No
50.22	Purell-type hand cleaner:	◯ Day	◯ Wk	◯ Mth	◯ <Mth	◯ No
50.23	Unscented laundry detergent:	◯ Day	◯ Wk	◯ Mth	◯ <Mth	◯ No
50.24	Scented laundry detergent:	◯ Day	◯ Wk	◯ Mth	◯ <Mth	◯ No
50.25	Fabric softener:	◯ Day	◯ Wk	◯ Mth	◯ <Mth	◯ No
50.26	Toilet bowl cleaner:	◯ Day	◯ Wk	◯ Mth	◯ <Mth	◯ No
50.27	Oven cleaner:	◯ Day	◯ Wk	◯ Mth	◯ <Mth	◯ No
50.28	Bathroom tile cleaner:	◯ Day	◯ Wk	◯ Mth	◯ <Mth	◯ No
50.29	Solvents (e.g. nail polish/paint remover):	◯ Day	◯ Wk	◯ Mth	◯ <Mth	◯ No

Figure S5. Questionnaire on household cleaning products use (Frequency of use score: 0 for never (not used), 1 for less than a month, 2 for monthly, 3 for weekly and 4 for daily).
